# Supplementary material for: Changes in Energy Status of Saccharomyces cerevisiae Cells during Dehydration and Rehydration
Source: Microorganisms. 2021 Feb 21;9(2):444. doi: 10.3390/microorganisms9020444 (PMC7926855; doi:10.3390/microorganisms9020444)
Supplement: Supplementary file 1 [file microorganisms-09-00444-s001.pdf]

Supplementary Materials

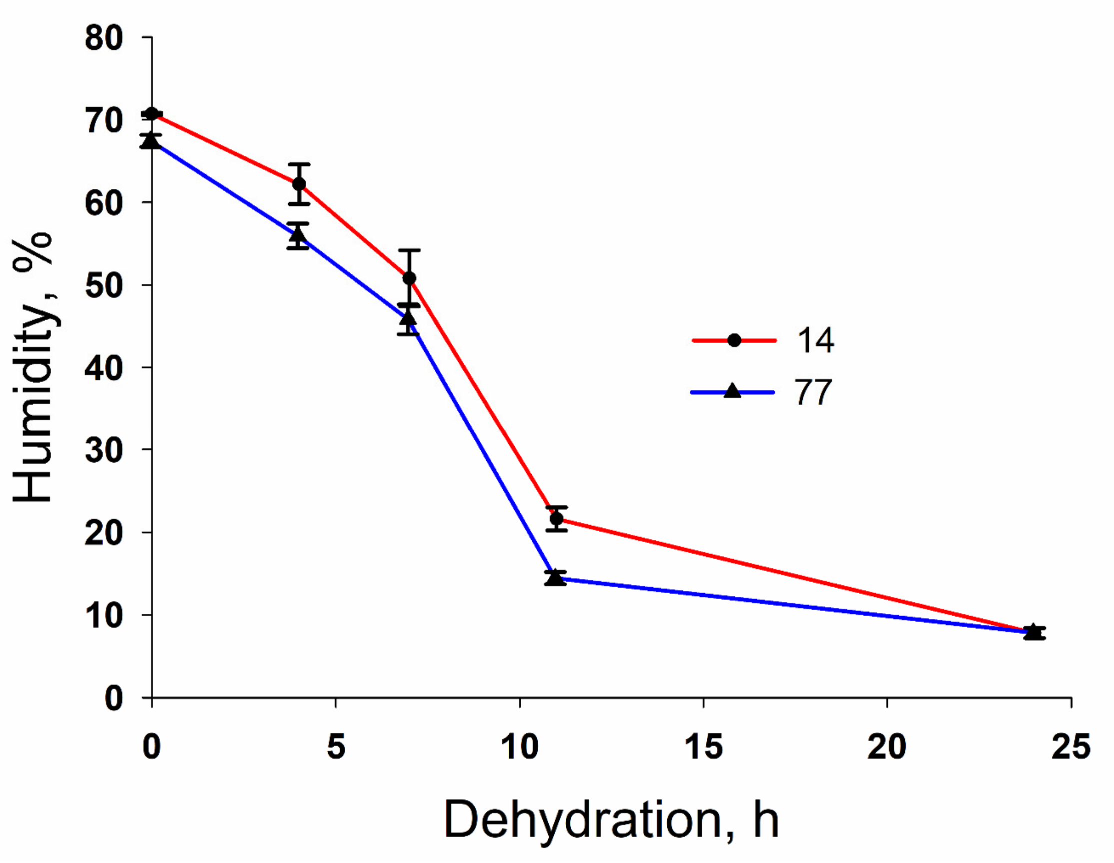

Figure S1. Changes in humidity of *S. cerevisiae* 14 and 77 cells during dehydration. The definite volumes of *S. cerevisiae* suspensions, containing 1.7x10^8 of cells (OD_{600} = 5.6) were aliquoted into 1.5 ml Eppendorf type tubes. The cells were washed twice with 100 mM sodium phosphate buffer, pH 7.0, and after the second wash the supernatant was carefully removed. Open tubes with the cell pellet were placed into an oven and dehydrated at 30 °C for 24 h. Tubes were weighed before and after the dehydration. Data are presented as Mean ± S.D. (n=3, three independent experiments).
